# Vγ9Vδ2 T cells expanded with vitamin C combined with HMBPP *in vitro* inhibit intracellular *Mycobacterium tuberculosis* growth

**DOI:** 10.3389/fcimb.2025.1533277

**Published:** 2025-04-30

**Authors:** Shuyan Liu, Guohui Xiao, Wanxin Liang, Su Zhang, Juan Liang, Teng Pan, Shaoxiang Lin, Xiuju Liu, Zhenwen Zhou, Guoliang Zhang

**Affiliations:** ^1^ National Clinical Research Center for Infectious Diseases, Guangdong Provincial Clinical Research Center for Tuberculosis, Shenzhen Third People’s Hospital, Southern University of Science and Technology, Shenzhen, China; ^2^ Longgang Maternity and Child Institute of Shantou University Medical College, Longgang District Maternity & Child Healthcare Hospital of Shenzhen City, Shenzhen, China

**Keywords:** *Mycobacterium tuberculosis*, Vγ9Vδ2 T cells, HMBPP, vitamin C, induction rate, effector function, inhibition

## Abstract

**Background:**

*Mycobacterium tuberculosis (Mtb)* presents a significant global health threat, and the existing treatments have notable limitations. Vγ9Vδ2 T cells activated by HMBPP can inhibit the growth of intracellular Mtb. Additionally, vitamin C (VC) promotes the differentiation and proliferation of related T cells. However, it remains uncertain whether VC can enhance the expansion of Vγ9Vδ2 T cells within PBMCs activated by HMBPP and rIL-2, and the underlying mechanism of the inhibitory effect of the expanded T cells on intracellular *Mtb* has not been elucidated.

**Methods:**

Venous blood was collected from healthy individuals, and PBMCs were subsequently isolated. In vitro, Vγ9Vδ2 T cells were selectively expanded with HMBPP, rIL-2, and VC. Flow cytometry was utilized to analyze the purities and phenotypes of Vγ9Vδ2 T cells, while cell counts were performed to determine the total number of viable cells. Magnetic bead sorting was employed to purify Vγ9Vδ2 T cells. *Mtb* strains were cultured, and macrophage infection models derived from THP1 cells were established. Co-culture experiments were conducted with *Mtb*-infected macrophages and Vγ9Vδ2 T cells, and the number of intracellular bacteria was quantified through CFU counting. The levels of cytokines were measured using the CBA method and flow cytometry. Statistical analysis was carried out using GraphPad Prism and SPSS software.

**Results:**

VC (70 μM) significantly enhances the expansion of Vγ9Vδ2 T cells within PBMCs during primary HMBPP activation in the presence of rIL-2, with higher induction rates and total cell proliferation. By day 14 of induction, Vγ9Vδ2 T cells expanded with HMBPP, VC, and rIL-2 exhibited the central memory (10-20%) and the effector memory phenotypes (75-90%). Furthermore, these expanded T cells effectively inhibited the growth of intracellular virulent *Mtb* strain (H37Rv) in a cell-contact-dependent manner. The inhibitory effect was associated with an up-regulated production of TNF-α and IFN-γ, and a down-regulated expression of IL-10 and IL-17A during *Mtb* infection.

**Conclusion:**

This study demonstrates that VC enhances the proliferative expansion of Vγ9Vδ2 T cells in PBMCs primarily stimulated with HMBPP and rIL-2. The expanded Vγ9Vδ2 T cells are capable of effectively inhibiting the growth of virulent H37Rv strain, likely through the secretion of TNF-α and IFN-γ. These findings provide a novel direction for tuberculosis treatment research.

## Graphical abstract

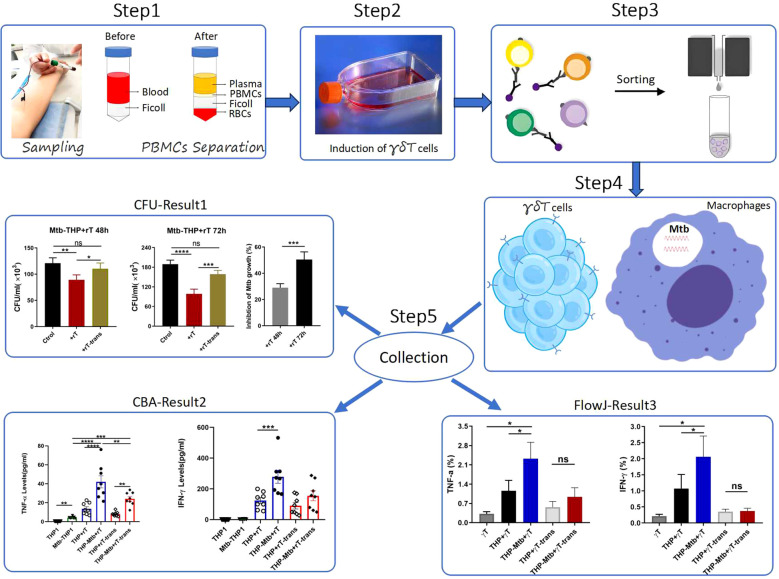

## 1 Introduction

*Mycobacterium tuberculosis* (*Mtb*), an ancient and highly successful intracellular pathogen, can cause various forms of tuberculosis, such as pulmonary, intestinal, and cerebral tuberculosis. Among these manifestations, pulmonary tuberculosis (TB) is the most widespread. It is recognized as a chronic infectious disease, and its situation is exacerbated by the rising incidence of co-infections and the emergence of drug resistance (Bourzac, 2014). The standard treatment for TB typically exceeds six months, and prolonged antibiotic use frequently leads to the development of multidrug-resistant tuberculosis (MDR-TB) (Bendre et al., 2021; Singh and Chibale, 2021). Furthermore, such treatments may significantly compromise the patient’s immune defenses (Bagcchi, 2023). Therefore, there is an urgent need to develop safe and effective therapeutic strategies for the management of tuberculosis.

Human γδ T cells, a subset of non-classical T cells, play a pivotal role in both innate and adaptive immune responses against infections, including those caused by *Mtb* and other pathogens (Shen et al., 2002; Yuan et al., 2009). These cells are divided into two main subpopulations: Vδ1 T cells, primarily located in mucosal tissues, and Vδ2 T cells, which are predominantly found in peripheral blood but can migrate to various sites. Research has demonstrated that the Vγ9Vδ2 T-cell subset is particularly crucial for inhibiting *Mtb* infection. This subset, unique to primates (including humans and non-human primates), constitutes 65–90% of total circulating human γδ T cells and expresses a Vγ9Vδ2-encoded T-cell receptor (TCR) (Chen, 2013).

Vγ9Vδ2 T cells recognize isoprenoid metabolites, including isopentenyl pyrophosphate (IPP) and (E)-4-hydroxy-3-methyl-but-2-enyl pyrophosphate (HMBPP), which was collectively termed phosphoantigens (Belmant et al., 1999). IPP is synthesized through two major pathways: the classical mevalonate (MVP) pathway and the alternative non-mevalonate (MEP) pathway. While IPP functions as an intermediate metabolite in both pathways, HMBPP is exclusively synthesized in the MEP pathway by specific microbes, including *Mycobacterium tuberculosis* and *Listeria monocytogenes* (Hintz et al., 2001). Under physiological conditions, Vγ9Vδ2 T cells can differentiate between pathogen-derived and host-derived prenyl pyrophosphates. The mevalonate pathway is prevalent in most eukaryotes, archaea, certain eubacteria, and the cytosol of plants. In contrast, the non-mevalonate pathway, also referred to as the 2-C-methyl-D-erythritol 4-phosphate (MEP) pathway, is present in most eubacteria, apicomplexan protozoa, cyanobacteria, and the chloroplasts of plants (Puan et al., 2007).

As an intermediate product of the MEP pathway and a microbe-specific metabolite, HMBPP is recognized as the most potent activator of Vγ9Vδ2 T cells, demonstrating an activity level that is 1000 fold higher *in vitro* compared to other physiological compounds (Hintz et al., 2001; Eberl et al., 2003; Reichenberg et al., 2003). Studies have demonstrated that HMBPP-specific Vγ9Vδ2 T cells can inhibit intracellular growth of Bacillus Calmette-Guérin (BCG) (Worku and Hoft, 2003), which depends on the production of HMBPP through a microbial-specific isoprenoid pathway (Puan et al., 2007). Chen ZW’s group demonstrated that transfer of Vγ9Vδ2 T cells to rhesus monkeys infected with BCG significantly enhances the anti-*Mtb* response (Shen et al., 2002). However, although HMBPP can activate Vγ9Vδ2 T cells *in vitro*, the induction rate reaches only 40-60% by the 14th day, even with the addition of rIL-2.

Vitamin C (L-ascorbic acid) is an essential nutrient for humans, serving as a potent antioxidant and a cofactor for a family of biosynthetic and gene regulatory enzymes. Extensive research has shown that vitamin C not only exhibits direct cytotoxicity toward tumor cells but also plays a critical role in supporting the cellular functions of both the innate and adaptive immune systems (Xu et al., 2021). Specifically, vitamin C enhances epithelial barrier function against pathogens and promotes the oxidant scavenging activity of the skin, thereby offering protection against environmental oxidative stress (Valacchi et al., 2015). Additionally, VC can facilitate the clearance of spent neutrophils from infection sites, reducing necrosis and potential tissue damage. The accumulation of VC in neutrophils enhances phagocytosis activity, stimulates the generation of reactive oxygen species, and promotes microbial killing (Boura et al., 1989; Goldschmidt, 1991). Although the role of VC in lymphocytes is less well-defined, some studies suggests it promotes the differentiation and proliferation of B cells, NK cells, regulatory T cells (Tregs), and dendritic cells derived from both murine and human hematopoietic progenitor cells (Anderson et al., 1980; Manning et al., 2013; Xu et al., 2021). VC deficiency is associated with impaired immunity and increased susceptibility to infections (Goldschmidt et al., 1988; Goldschmidt, 1991). Conversely, infections significantly deplete VC levels due to heightened inflammatory responses and increased metabolic demands. Furthermore, VC supplementation has been shown to prevent and treat respiratory and systemic infections (Carr and Maggini, 2017).

Kouakanou et al. demonstrated that VC enhances the proliferative expansion and effector function of purified human Vγ9Vδ2 T cells, as well as 14-day-expanded Vγ9Vδ2 T-cell lines restimulated with zoledronate (ZOL) or synthetic phosphoantigens (pAgs) (Kouakanou et al., 2020). Furthermore, VC was found to increase the proliferation of Vγ9Vδ2 T cells within Peripheral blood mononuclear cells (PBMCs) selectively activated with Zol, which subsequently enhanced the tumor-killing activity of these γδ cells expanded *in vitro* (Xu et al., 2021). Specifically, VC reduced apoptosis in Vγ9Vδ2 T cells and increased cytokine production during both primary activation and subsequent pAg restimulation, and also augmented oxidative respiration and glycolysis in γδ T cells during pAg restimulation (Kouakanou et al., 2020).

In this study, we investigated whether VC could significantly enhance the proliferative expansion of Vγ9Vδ2 T cells within PBMCs primarily activated with HMBPP and rIL-2. Furthermore, we explored the ability and underlying mechanism of Vγ9Vδ2 T cells expanded *in vitro* with HMBPP, VC, and rIL-2 to inhibit the growth of intracellular *Mtb*. This research provides valuable theoretical insights for the development of novel *Mtb* vaccines.

## 2 Materials and methods

### 2.1 Venous blood collection, PBMC isolation and ethics

Healthy individuals aged 20 to 40 years were recruited for this study after obtaining informed consent. Prior to blood collection, participants underwent routine blood tests to confirm that all blood parameters were within normal ranges and to exclude symptoms such as fever, cold or diarrhea. Additionally, participants were screened for infectious diseases (e.g., HIV, HBV, HCV) and underlying conditions (e.g., hypertension, diabetes). A total of 50–100 ml of venous blood was collected from each participant and stored in blood collection bags. PBMCs were isolated using the Ficoll-Paque density gradient centrifugation method (Brosseron et al., 2015). If immediate separation was not feasible, blood samples were stored at 4°C for no longer than 6 hours.

The study protocol was approved by the Regional Ethics Committee of Shenzhen Third People’s Hospital (Approval ID: SZLY2021049). All participants provided written informed consent in accordance with the Declaration of Helsinki.

### 2.2 *Ex vivo* selective expansion of Vγ9Vδ2 T cells by HMBPP, rIL-2 and VC in PBMC cultures

Freshly isolated PBMCs were cultured at a density of 3.0 × 10^6^ cells/ml in RPMI 1640 medium (Gibco, C11875500BT) supplemented with 10% FBS (Gibco,10099-141) and 1% penicillin-streptomycin (PS) (Gibco, 15140122) and varying concentrations of HMBPP (50nM, 100nM and 200nM) (Sigma, 95098), 100 IU/ml rIL-2 (Beijing Four Rings Bio-Pharm Co. S10970016), and, where indicated, 70 µM L-ascorbic acid (VC) (Sigma, A4403). On day 3, the medium and cytokines were refreshed, excluding HMBPP. Cells were collected using 1× PBS (Gibco, C10010500BT), centrifuged at 600–800 rpm for 7 minutes, and resuspended in fresh medium to achieve a density of 2.0-2.5 × 10^6^ cells/ml. On day 5, the medium was replaced again, maintaining a centrifugal force of 800 rpm and a cell density of 2.0 × 10^6^ cells/ml. From day 7 onwards, the medium was changed every 2–3 days, maintaining a cell density of 1.0-2.0 × 10^6^ cells/ml. Cells were cultured for a total of 14–21 days, with the total cell count and Vγ9Vδ2 T-cell purity recorded at each medium change.

### 2.3 Flow cytometry and cell counts

Induced Vɣ9Vδ2 T cells were stained with fluorochrome-conjugated monoclonal antibodies targeting CD3-APC-H7 (SK7, 560176), CD3-APC-CY7 (SK7, 341110), TCRVδ2-PE (B6, 555739), TCRVδ2-FITC (B6, 555738), CD27-APC (M-T271, 558664), CD45RA-FITC(HI100, 555488), CD4-PE-CY7 (SK3, 557852), CD4-PerCP (L200, 550631), CD8-APC (RPA-T8, 555369), IFN-γ-APC (4S.B3, 551385), and TNF-α-PE-Cy7(MAb11, 557647) (all from BD Biosciences). The staining protocol followed our previous study (Liu et al., 2016). Briefly, expanded cells were harvested, washed with PBS, and stained with surface antibodies for 30 minutes at 4°C in the dark. For intracellular cytokine staining, cells were treated with Brefeldin A (BFA, eBioscience,00-4506-51), monensin (BD, 554714), and ionomycin for 6 hours prior to staining. Cells were fixed and permeabilized using BD fixation/permeabilization solution (BD, 554714) and stained with intracellular cytokine antibodies. After staining, cells were washed twice with PBS and resuspended in PBS for flow cytometer analysis. If immediate analysis was not possible, cells were fixed with 4% paraformaldehyde and analyzed as soon as possible.

Vɣ9Vδ2 T-cell ratio within total T cells was analyzed using anti-human CD3-APC-H7, TCR Vδ2-PE, CD27-APC and CD45RA-FITC to identify the cell subtypes (Meraviglia et al., 2010). For TNF-α and IFN-γ staining, antibodies against CD3-APC-Cy7, TCRVδ2-PE, TNF-α-PE-Cy7 and IFN-γ-APC were used. The absolute number of viable Vγ9Vδ2 T cells were quantified using the Cell-Meter K2 after excluding dead cells.

### 2.4 Magnetic bead sorting of Vɣ9Vδ2 T cells

After 14 to19 days of induction, Vɣ9Vδ2 T cells with a purity exceeding 70% were purified using anti-human TCR Vδ2-PE antibody and anti-PE magnetic beads (Miltenyi Biotec, 130-048-801). Briefly, cells were collected, washed with PBS and stained with anti-human TCR Vδ2-PE antibody at a ratio of 10^7^ cells to 10 µL antibody. After incubation at 4°C for 15 minutes, cells were washed and incubated with anti-PE microbeads for an additional 15 minutes. Cells were then resuspended in sorting buffer (Miltenyi Biotec, 130-091-222) supplemented with 0.5% bovine serum albumin (BSA) stock solution (Miltenyi Biotec, 130-091-376) and sorted using a magnetic column. The purified Vɣ9Vδ2 T-cell population exhibited over 98% viability, as confirmed by flow cytometry. In some cases, anti-human TCRγ/δ microBeads (Miltenyi Biotec, 130-050-701) were used to prevent overlap with mCherry or PE fluorescence.

### 2.5 Bacterial strains and culture conditions

The wild-type *Mtb* strain (H37Rv, virulent strain) and the avirulent strain expressing mCherry fluorescence (H37Ra-mCherry) were cultured at 37°C in Middlebrook 7H9 broth or 7H10 agar supplemented with 10% (vol/vol) oleic acid–albumin–dextrose–catalase (OADC, BD, 211886), 0.5% glycerol and 0.05% (v/v) tyloxapol. Bacterial cultures required approximately 14–21 days to reach the logarithmic growth phase or form visible colonies on agar plates.

### 2.6 Induction of human primary macrophage and *Mtb* infection

PBMCs from healthy donors were counted and seeded in 10-cm cell culture dishes at a density of 2-3× 10^6^ cells/ml in RPMI 1640 medium supplemented with 10% FBS, 1% PS and HEPES. Macrophage differentiation was induced by adding macrophage colony-stimulating factor (M-CSF, R&D Systems, 216-MC-025) at a final concentration of 10 ng/ml for 7 days. Half of the medium was replaced every 3–4 days, maintaining the same M-CSF concentration. On day 7, non-adherent cells were removed, and adherent cells were washed twice with 1× PBS. Cells were detached using 0.05% trypsin (0.5–1 ml/dish) at 37°C incubator for 10-15min, with gentle shaking every 2–3 minutes. The cells were repeatedly pipetted up and down using a 3-ml Pasteur pipette until the complete detachment was achieved. Cells were collected, centrifuged at 1500 rpm, washed with PBS, and counted.

A portion of the cells was stained to assess the induction efficiency and purity of macrophages by measuring the percentage of CD14-positive cells (CD14+). For infection experiments, 2 × 10^5^ cells were seeded in a 24-well plate. After adherence, macrophages were infected with H37Ra-mCherry strain at multiplicities of infection (MOIs) of 10 and 20. Following a 4-hour adsorption period, infected macrophages were co-incubated with purified Vɣ9Vδ2 T cells. After 48 h, cells were trypsinized, and the infection rate was determined by flow cytometry as the percentage of red fluorescent macrophages relative to the total macrophages population.

### 2.7 *THP-1*-derived macrophages differentiation and *Mtb* infection

*THP-1* cells (ATCC) were cultured in RPMI 1640 medium supplemented with 10% FBS, 1% HEPES and 1% PS. For differentiation, *THP-1* cells were seeded at 4.0×10^5^ cells/ml (2.0×10^5^ cells/well in 24-well plates) and treated with 50 ng/ml phorbol 12-myristate 13-acetate (PMA, P-8139, Sigma) for 24h. The medium was then replaced with PMA-free medium, and cells were allowed to recover for an additional 24h. On day 3, *THP-1*-derived macrophages were infected with the H37Ra-mCherry strain at a MOI of 10. The proportion of fluorescent cells, indicating the infection rate, was assessed by flow cytometry at 48 h and 72 h post-infection. For quantitative analysis, *THP-1*-derived macrophages were infected with the H37Rv strain at MOI of 10, and bacterial loads were determined by serial dilution and colony-forming unit (CFU) counting.

### 2.8 Co-culture of *Mtb*-infected macrophages and Vɣ9Vδ2 T cells, CFU counting

Co-culture experiments were performed under two conditions: (1) direct contact between Vγ9Vδ2 T cells (effector cells) and *Mtb*-infected macrophages (target cells), and (2) indirect contact using 0.4-µm transwell chambers (Corning, Cat. 3470), allowing only supernatant exchange. Six experimental groups were established: (1) uninfected *THP-1*-derived macrophages (THP1), (2) H37Rv-infected *THP-1*-derived macrophages (Mtb-THP1), (3) H37Rv-infected *THP-1*-derived macrophages co-cultured with Vγ9Vδ2 T cells (THP-Mtb+rT), (4) uninfected *THP-1*-derived macrophages co-cultured with Vγ9Vδ2 T cells (THP+rT), (5) indirect co-culture of uninfected *THP-1*-derived macrophages with Vγ9Vδ2 T cells (THP+rT-trans), and (6) indirect co-culture of H37Rv-infected *THP-1*-derived macrophages with Vγ9Vδ2 T cells (THP-Mtb+rT-trans).

After 4 h of H37Rv infection, non-adherent bacteria were removed by washing twice with 1× PBS. Vγ9Vδ2 T cells were added at an effector-to-target ratio of 10:1. At 48 and 72 h post-infection, supernatants were collected and stored at −80°C. Cells were lysed with 0.1% SDS, and intracellular bacteria were quantified by serial dilution and CFU counting on 7H10 agar plates. Colonies were enumerated after 14–21 days of incubation.

### 2.9 Measuring cytokine production by CBA method

Following macrophages infected with *Mtb*, macrophages were co - cultured with purified Vγ9Vδ2 T cells for 48 h and 72 h respectively. Cell culture supernatants were then collected for subsequent analysis. A 50 μl aliquot of each supernatant was transferred into separate tubes and stored at -80°C for further analysis. The levels of inflammatory factors and cytokines associated with Vɣ9Vδ2T cell function in the supernatants of the six cell infection groups (as described in the Method 8) were quantified using the Cytometric Bead Array (CBA) technique. Specifically, the LEGENDplex™ multi-analyte flow assay kit (human CD8/NK panel,13-plex, Cat. No.740267, BioLegend) was employed to detect the concentrations of 13 factors, including IL-2, IL-4, IL-17A, IL-6, IL-10, TNF-α, IFN-γ, Fas, FasL, granzyme A (GrzA), granzyme B (GrzB), perforin (PRF) and granulysin (GNLY). All experimental procedures were performed in strict compliance with the manufacturer’s instructions. Following the completion of the assay, C0-C8 standard curves for each factor were generated to validate the multiplexed concentration measurements. These curves confirmed that *R^2^ value* for each cytokine standard curve exceeded 0.99 (**Supplementary Figure S3**). Using the standard curve derived from known protein concentrations, the concentrations of the 13 factors in the supernatants of the test samples were accurately determined.

### 2.10 Statistical analysis

Statistical analyses were performed using GraphPad Prism (version 8.0.1). Comparisons between groups were performed using a paired two-tailed Student’s t-test or one-way ANOVA followed by Bonferroni correction for multiple comparisons. Pearson correlation analysis was conducted using SPSS (version 13.0). A p-value < 0.05 was considered statistically significant. All data are available from the corresponding author upon reasonable request.

## 3 Results

### 3.1 Vitamin C enhances the induction and expansion of high-purity Vγ9Vδ2 T cells from healthy human PBMCs during HMBPP primary activation

**Figure 1:**
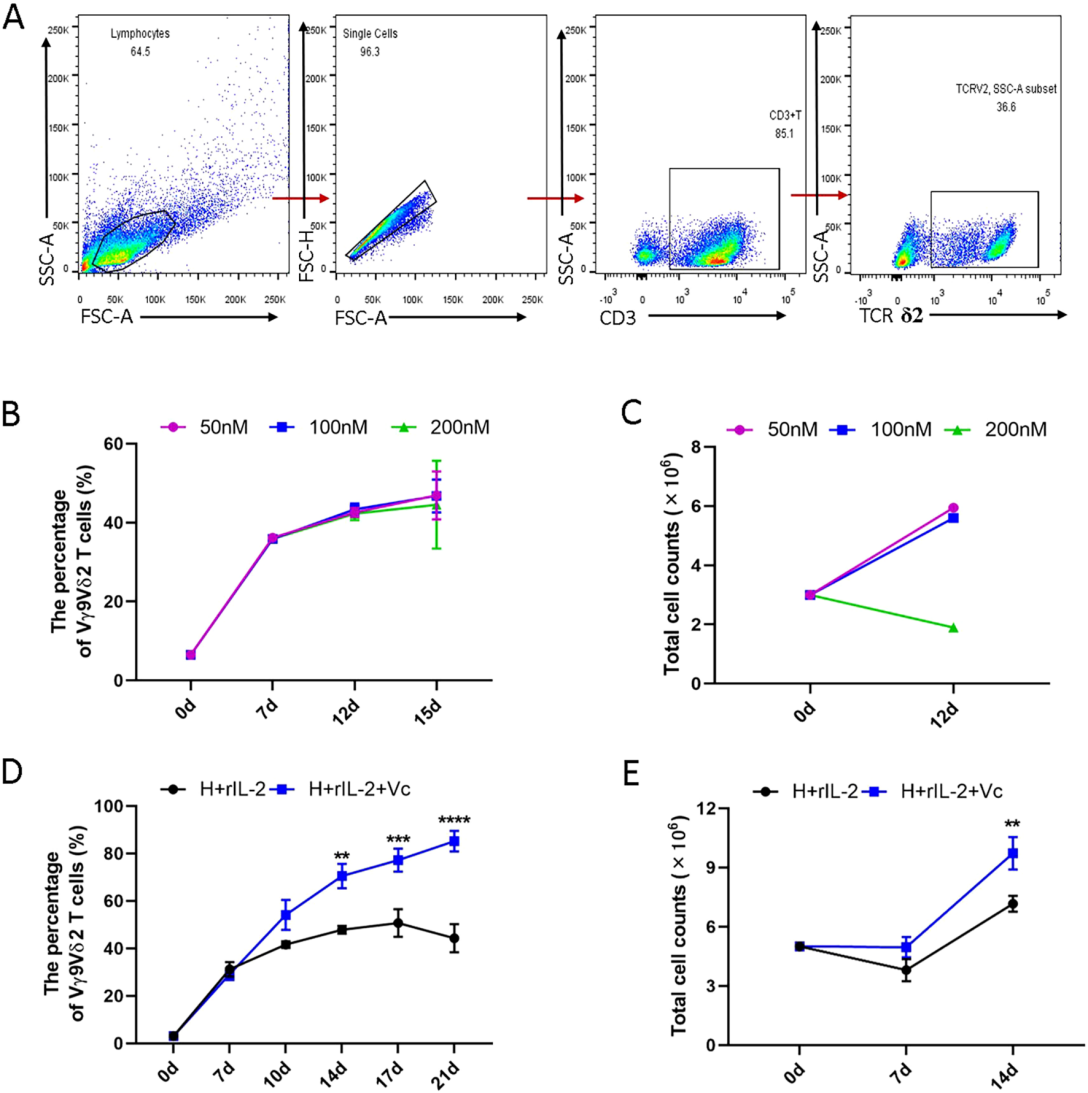
Comparison of the proliferation of Vγ9Vδ2 T cells induced and expanded with or without VC in combination with HMBPP and rIL-2 *in vitro*. Healthy donor PBMCs were activated with varying concentrations of HMBPP (50 nM, 100 nM, 200 nM) for three days, supplemented simultaneously with rIL-2. The culture medium was refreshed every 2–3 days, after which HMBPP was removed. Cells were harvested to evaluate the induction rate by flow cytometry and to quantify total cell numbers using an automated cell counter. Induced cells were stained with surface-specific antibodies to determine the proportion of Vγ9Vδ2 T cells within the CD3+ T cell population. **(A)** Representative gating strategy and flow cytometry plots of Vγ9Vδ2 T cells in PBMCs. **(B)** Induction rates of Vγ9Vδ2 T cells and **(C)** total number of proliferating cells activated by varying concentrations of HMBPP (200 nM, 100 nM and 50 nM) in the presence of rIL-2. Data are presented as mean ± SEM from two independent experiments pooled from 2 healthy dornors. **(D)** Induction ratio of Vγ9Vδ2 T cells in PBMCs and **(E)** total number of proliferating cells activated and expanded with HMBPP (50 nM) and rIL-2, with or without VC (70 μM). Data are presented as mean ± SEM from three independent experiments pooled from 7 healthy dornors. Statistical analyses were performed using one-way ANOVA followed by Bonferroni multiple comparison test. **P<0.01, ***P<0.001, ****P<0.0001.

Previous studies have demonstrated that human PBMCs can differentiate into a substantial population of Vγ9Vδ2 T cells upon HMBPP induction *in vitro* (Dunne et al., 2010). To further investigate this phenomenon, we established an optimized experimental platform for the robust proliferation of HMBPP-activated Vγ9Vδ2 T cells. In this study, PBMCs were treated with varying concentrations of HMBPP (50 nM, 100 nM, and 200 nM) to induce of Vγ9Vδ2 T cell differentiation, supplemented with rIL-2 to expand HMBPP-specific Vγ9Vδ2 T cells. The gating strategy is illustrated in **Figure 1A**. Consistent with prior reports, we observed that the 200nM concentration resulted in reduced cell proliferation, whereas the 50 nM and 100 nM concentrations effectively promoted the differentiation and proliferation of Vγ9Vδ2 T cells, leading to a stable increase in both cell purity (≥90%) and total cell numbers (**Figures 1B, C**, p < 0.05). Based on these findings, we selected the 50 nM HMBPP concentration for subsequent functional experiments.

It has been reported that vitamin C enhances the host immune response. Specially, under the stimulation of ZOL or other artificially synthesized pAgs, VC promotes the proliferation and effector functions of purified Vγ9Vδ2 T cells, as well as the restimulation of 14-day-expanded γδ T cells (Kouakanou et al., 2020). We hypothesized that vitamin C could similarly enhance the proliferation and effector function of γδ T cells in PBMCs stimulated with primary HMBPP. To test this hypothesis, we compared the induction efficiency of Vγ9Vδ2 T cells using two protocols: (1) HMBPP+rIL-2 and (2) HMBPP+VC (50nM) +rIL-2. Our results demonstrated that the HMBPP, VC, and rIL-2 protocol significantly outperformed the traditional HMBPP+rIL-2 method in terms of induction purity (p < 0.01) and cell proliferation rate (p < 0.001, **Figures 1D, E**).

In the presence of VC, the induction ratio of Vγ9Vδ2 T cells reached 30-40% by day 7 and exceeded 70% by day 14, with a peak induction ratio of 75.2 ± 3.1% on day 17. In contrast, the traditional method exhibited a decline after day 14, reaching only 45.3 ± 2.8% by day 17 (**Figure 1D**, p < 0.01). Furthermore, VC significantly accelerated total cell counts over the induction period, with cell numbers on day 14 being 2–4 times higher than those in the HMBPP+rIL-2 group (**Figure 1E**, p < 0.01). These findings suggest that vitamin C not only enhances the induction efficiency of Vγ9Vδ2 T cells but also sustains their proliferation over an extended period.

### 3.2 Phenotypic characterization of Vγ9Vδ2 T cells expanded with HMBPP, VC, and rIL-2

**Figure 2:**
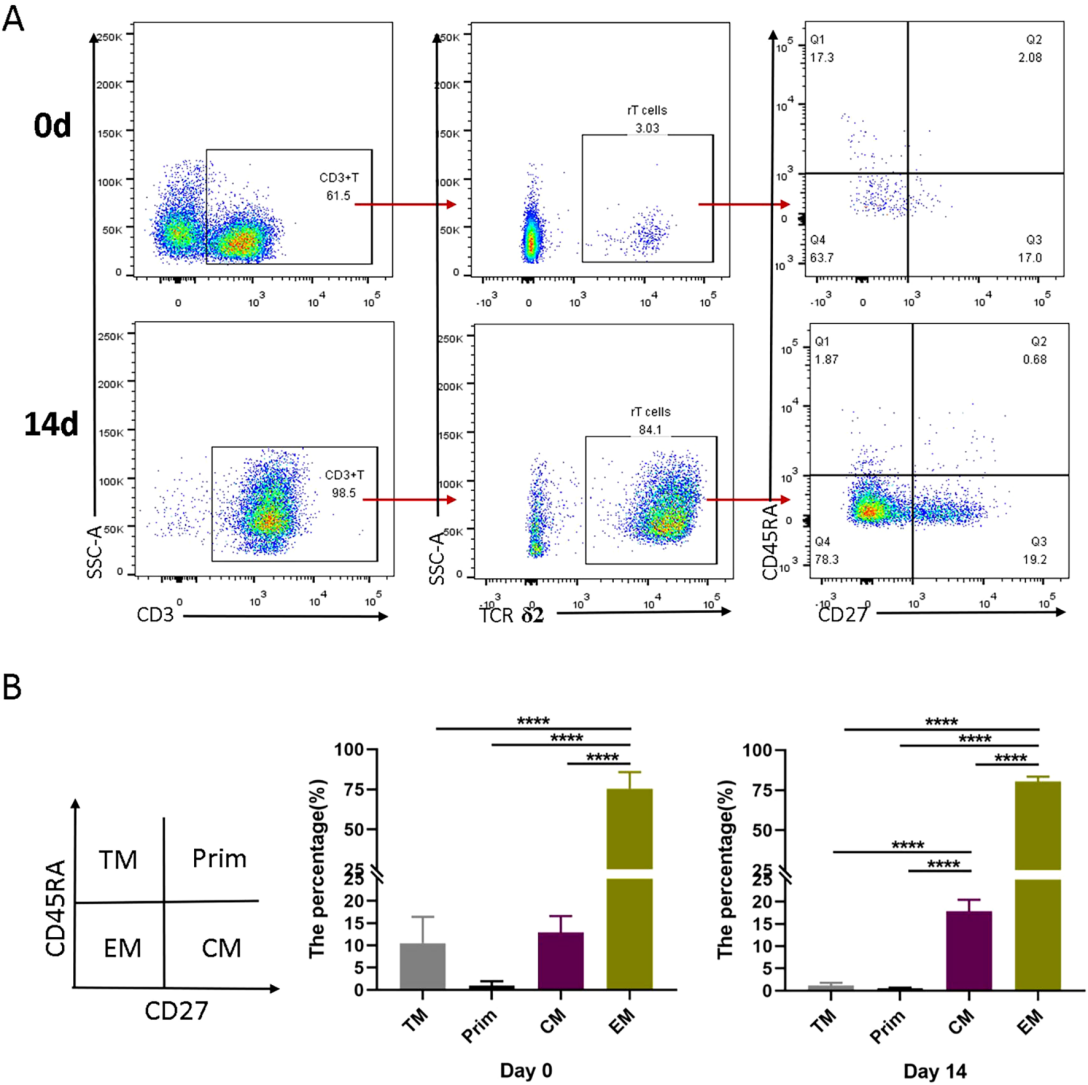
Differentiation subsets of Vγ9Vδ2 T cells induced *in vitro* by VC in combination with HMBPP and rIL-2. Healthy donor PBMCs were activated and expanded with HMBPP, VC, and rIL-2 for 14 days. The phenotypic characterization of Vγ9Vδ2 T cells was performed by staining with surface antibodies, including anti-CD3-APC-H7, anti-TCRδ2-PE, anti-CD45RA-FITC and anti-CD27-APC. **(A)** Representative flow cytometry plots illustrating the gating strategy and the subsets of Vγ9Vδ2 T cell in PBMCs at day 0 and day 14. **(B)** Comparison of the proportion of different Vγ9Vδ2 T cell subsets at day 0 and day 14 of induction. Subsets were defined as follows: terminally differentiated cells (CD45RA^+^CD27^−^), naive cells (Prim, CD45RA^+^CD27^+^), effector memory T cells (EM, CD45RA^−^CD27^−^) and central memory cells (CM, CD45RA^−^CD27^+^). Representative data are from three independent experiments, each performed using PBMCs from a healthy donor (n=3). Statistical analyses were conducted using one-way ANOVA followed by Bonferroni multiple comparison test. ****P<0.0001.

To further characterize the expanded Vγ9Vδ2 T cells, we analyzed their phenotypic profiles using flow cytometry. Comparing the γδ T cell phenotypes in initial PBMCs and those after 14 days of induction, we found that the expanded Vγ9Vδ2 T cells were predominantly composed of effector memory T cell (EM, CD45RA^-^CD27^-^, 75-90%) and central memory T cells (CM, CD45RA^-^CD27^+^, 10-20%) (**Figures 2A, B**). Terminally differentiated cells (TM, CD45RA^+^CD27^-^) and naive cells (Prim, CD45RA^+^CD27^+^) accounted for ≤5% and ≤1%, respectively (**Figure 2B**). These results indicate that the HMBPP + vitamin C + rIL-2 protocol preferentially induces a memory phenotype in Vγ9Vδ2 T cells, which may enhance their functional capacity against intracellular pathogens.

### 3.3 Vγ9Vδ2 T cells expanded with HMBPP, VC, and rIL-2 inhibit intracellular *Mtb* growth

Previous studies have demonstrated that BCG-specific or HMBPP-specific Vγ9Vδ2 T cells can reduce the viability of *BCG or Mtb* (Dieli et al., 2000; Spencer et al., 2013; Yang et al., 2019). However, these cells were typically expanded using *BCG*, HMBPP combined with IL-2, IL-12 or IL-15. In this study, we investigated whether Vγ9Vδ2 T cells expanded with HMBPP, VC, and rIL-2 could inhibit the growth of intracellular *Mtb*.

Primary macrophages were infected with the attenuated H37Ra-mCherry strain, and after 7 days of PBMC induction, CD14-positive macrophages exceeded 95% purity (**Supplementary Figure S1B**). Macrophages were infected with MOIs of 10 and 20, and unbound bacteria were removed after 4 hours of adsorption. Vγ9Vδ2 T cells, isolated using magnetic bead sorting (**Supplementary Figure S1A**), were co-cultured with infected macrophages at an effector-to-target ratio of 10:1. Flow cytometry analysis revealed that the infection rates were 35.5% (MOI = 10) and 54.3% (MOI = 20) at 48h and 72h post-infection, respectively. The addition of Vγ9Vδ2 T cells significantly reduced these rates to 13.5% and 16%, respectively (p < 0.001), suggesting their potent inhibitory effect on intracellular *Mtb* growth (**Supplementary Figure S1C**).

**Figure 3:**
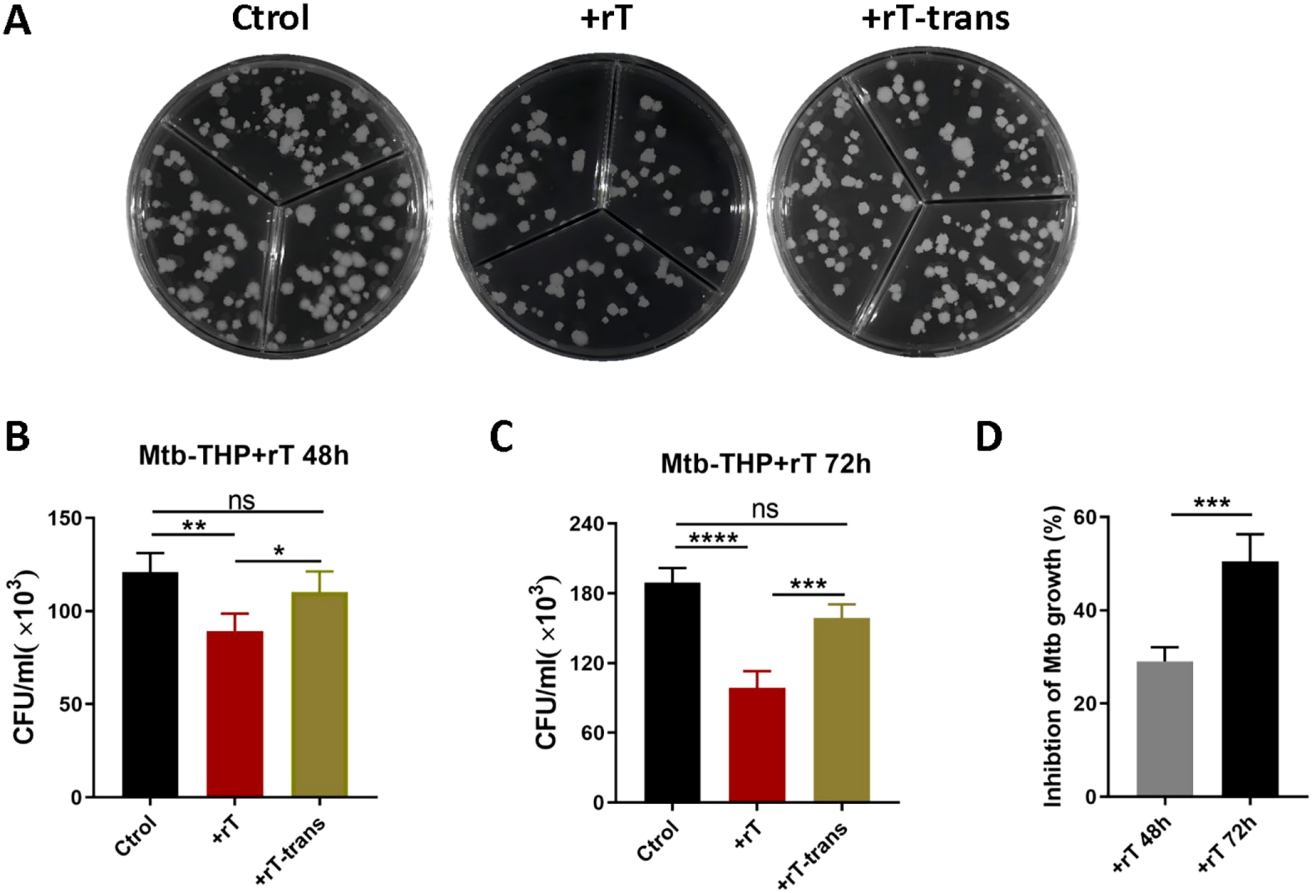
Vγ9Vδ2 T cells expanded with HMBPP, VC, and rIL-2 efficiently inhibit the growth of intracellular *Mtb*. *THP-1*-derived macrophages were infected with virulent H37Rv strain at MOI of 10 for 4h. Subsequently, Vγ9Vδ2 T cells expanded using HMBPP, VC, and rIL-2 were added to the culture medium at an effector-to-target cell ratio of 10:1. The Vγ9Vδ2 T cells were either placed in direct contact with *Mtb*-infected macrophages or separated using 0.4μM transwell chambers for indirect contact. At 48h and 72h post-infection, co-cultured cells were harvested and lysed with 0.1% SDS to analyze the intracellular *Mtb* infection rate. **(A)** Comparison of colony growth on agar plates of intracellular *Mtb* in *Mtb*-infected *THP-1* macrophages. Comparison of the direct contact group and the indirect contact group at **(B)** 48h and **(C)** 72h post-infection. **(D)** Inhibition rates of Vγ9Vδ2 T cells on *Mtb* growth at 48h and 72h post-infection, using T cells expanded with HMBPP, VC, and rIL-2. Ctrol: *Mtb*-infected *THP-1*-derived macrophages. +rT: direct contact group of *Mtb*-infected *THP-1*-derived macrophages with Vγ9Vδ2 T cells, +rT-trans: indirect contact group of *Mtb*-infected *THP-1*-derived macrophages with Vγ9Vδ2 T cells. Data are presented as mean ± SEM from three independent experiments using PBMCs pooled from 8 healthy donors. Statistical analyses were performed using t-test and one-way ANOVA followed by Bonferroni multiple comparison test. *P<0.05, **P<0.01, ***P<0.001, ****P<0.0001. ns, no significant.

Similar experiments using *THP-1*-derived macrophages confirmed these findings. Similar experiments using *THP-1*-derived macrophages confirmed these findings. Confocal microscopy revealed that Vγ9Vδ2 T cells aggregated around H37Ra-infected macrophages (**Supplementary Figure S2A**), and flow cytometry showed a significant decrease in H37Ra infection rates (**Supplementary Figure S2B**). To assess the effect on virulent strain, *THP-1*-derived macrophages were infected with H37Rv strain (MOI=10) and co-cultured with purified Vγ9Vδ2 T cells. A transwell system was used to separate effector and target cells. Results indicated that direct contact between Vγ9Vδ2 T cells and macrophages significantly inhibited the growth of intracellular *Mtb* (**Figures 3A, B**, p < 0.001), whereas indirect contact had minimal effect (**Figures 3A, C**). The inhibitory effect was more pronounced at 72h compared to 48h (**Figure 3D**, p < 0.01), suggesting a time-dependent enhancement of Vγ9Vδ2 T cell-mediated anti-mycobacterial activity.

### 3.4 Vγ9Vδ2 T cells expanded with HMBPP, VC, and rIL-2 downregulated IL-10, but upregulated TNF- α during *Mtb* infection

To further elucidate the immunomodulatory effects of Vγ9Vδ2 T cells, we investigated their impact on the inflammatory response during *Mtb* infection. Previous studies have reported a significant increase in the serum levels of the inflammatory factor IL-6 in tuberculosis patients (Qiu et al., 2008; Coulter et al., 2017). Therefore, we aimed to determine whether Vγ9Vδ2 T cells could modulate macrophage-mediated inflammatory response.

**Figure 4:**
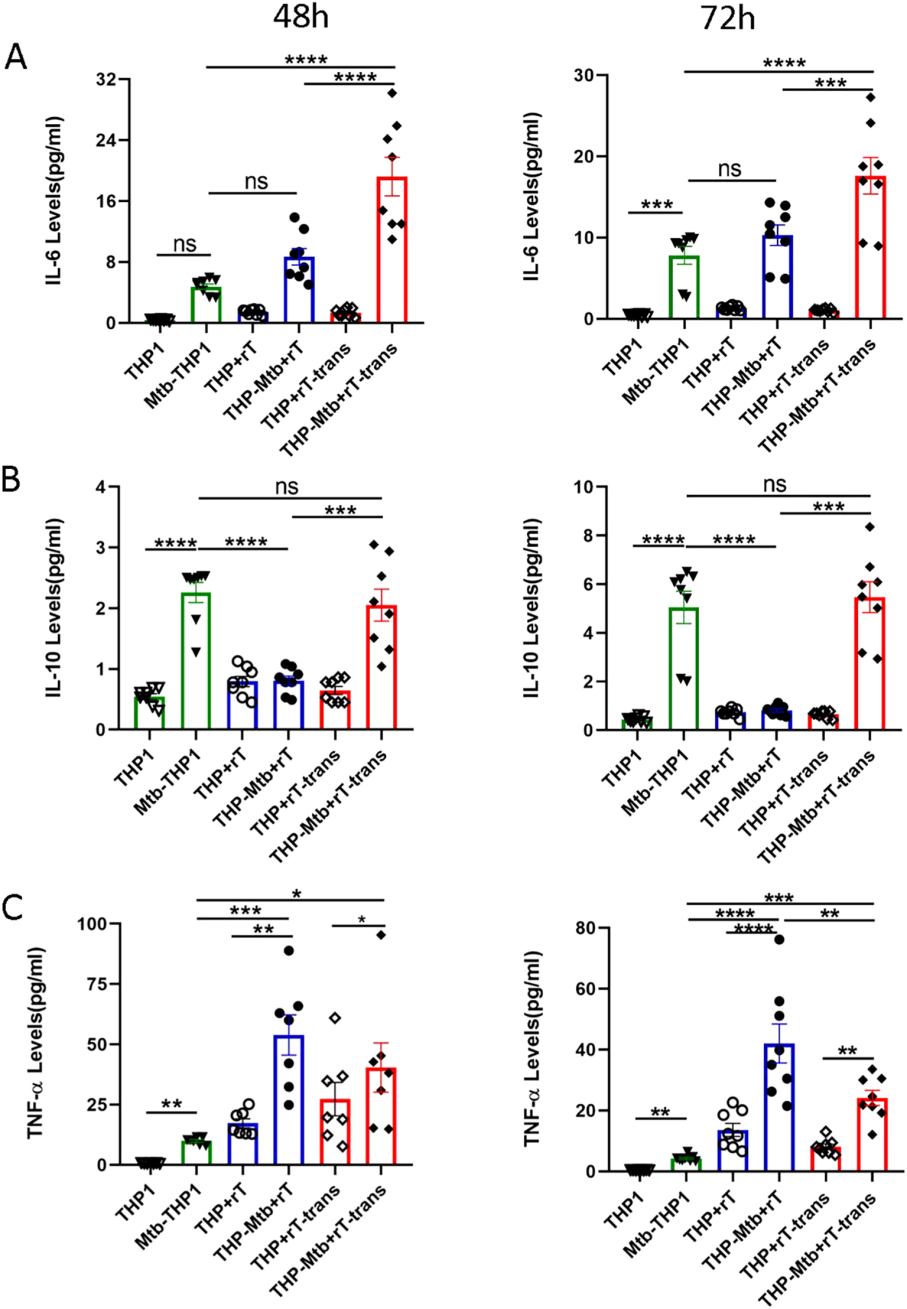
Vγ9Vδ2 T cells expanded with HMBPP, VC, and rIL-2 downregulate IL-10 levels but upregulate TNF- α expression during mycobacterial infection. Supernatants from six cell infection groups were collected to quantify inflammatory cytokine levels using a cytometric bead array. These groups included: *THP-1*-derived macrophages (THP1), *Mtb*-infected macrophages (Mtb-THP1), direct contact group of *THP-1*-derived macrophages with Vγ9Vδ2 T cells (THP+rT), direct contact group of *Mtb*-infected *THP-1*-derived macrophages with Vγ9Vδ2 T cells (THP-Mtb+rT), indirect contact group of *THP-1*-derived macrophages with Vγ9Vδ2 T cells using transwell chambers (THP+rT-trans), and indirect contact group of *Mtb*-infected *THP-1*-derived macrophages with Vγ9Vδ2 T cells using transwell chambers (THP-Mtb+rT-trans). Representative expression levels of **(A)** IL-6, **(B)** IL-10 and **(C)** TNF-α in the six experimental groups at 48h and 72h post-infection. Data are presented as mean ± SEM from three independent experiments pooled from 6 healthy donors. Statistical analyses were performed using one-way ANOVA followed by the Bonferroni multiple comparison test. *P<0.05, **P<0.01, ***P<0.001, ****P<0.0001.

We quantified the expression of inflammatory factors in the supernatant of *Mtb*-infected *THP-1*-derived macrophages using CBA assay. Standard curves were constructed for each cytokine to ensure accurate quantification (**Supplementary Figure S3**). Our results demonstrated that *Mtb* infection significantly upregulated the expression of both IL-6 and IL-10 in *THP-1*-derived macrophages. Direct co-culture with Vγ9Vδ2 T cells downregulated IL-10 levels (p < 0.01) but had no significant effect on IL-6 levels (**Figures 4A, B**). In contrast, indirect co-culture with Vγ9Vδ2 T cells upregulated IL-6 levels (p < 0.05) without affecting IL-10 levels, with IL-6 levels significantly higher in the indirect contact group compared to the direct contact group (**Figures 4A, B**, p < 0.01)).

TNF-α, a crucial pro-inflammatory cytokine secreted by activated macrophages, T cells, and NK cells. TNF-α not only promotes the inflammatory response but also enhances the proliferation and cytotoxic functions of T cells. Our experiment indicated that TNF-α levels were significantly upregulated in the presence of Vγ9Vδ2 T cells, particularly in direct co-culture group (**Figure 4C**, p < 0.0001)). These findings suggest that Vγ9Vδ2 T cells exert distinct immunomodulatory effects depending on their interaction mode with infected macrophages, potentially enhancing anti-mycobacterial immunity through TNF-α upregulation and IL-10 downregulation.

### 3.5 Vγ9Vδ2 T cells expanded with HMBPP, VC, and rIL-2 suppresses intracellular *Mtb* growth via downregulation of IL-17A and upregulation of IFN-γ

To further elucidate the mechanisms underlying the anti-mycobacterial activity of Vγ9Vδ2 T cells, we investigated the effector molecules involved in their inhibitory effects on intracellular *Mtb* growth. Using the CBA assay, we quantified the expression of cytokines associated with the differentiation and activation of Vγ9Vδ2 T cell subpopulations.

IL-4, a marker for T cell subpopulations differentiation, was minimally expressed by Vγ9Vδ2 T cells, however, with no significant difference observed upon exposure to conditioned medium from *Mtb*-infected macrophages (**Figure 4A**). In contrast, Vγ9Vδ2 T cells expanded with this protocol significantly downregulated the expression of IL-17A during inhibition of intracellular *Mtb* growth (**Figure 4B**). This observed downregulation of IL-17A was independent of direct cell-to-cell contact, indicating a cytokine-mediated regulatory mechanism.

Additionally, the Vγ9Vδ2 T cells expanded with HMBPP, VC, and rIL-2 significantly upregulated the levels of IFN-γ (p < 0.001), a key cytokine known to potentiate anti-mycobacterial immunity. Notably, the IFN-γ level was strictly dependent on direct cell contact, as indirect co-culture conditions using Transwell systems failed to elicit significant IFN-γ upregulation (p > 0.05, **Figure 4C**). Collectively, these findings indicate that IFN-γ mediates the direct anti-mycobacterial effector function of Vγ9Vδ2 T cells, while IL-17A downregulation may contribute to dampening excessive inflammation during *Mtb* infection.

### 3.6 Vγ9Vδ2 T cells expanded with HMBPP, VC, and rIL-2 do not increase direct cytotoxicity-related factor expression during *Mtb* infection

The effector functions of Vɣ9Vδ2 T cells against tumor cells or infected cells are commonly mediated by direct cytotoxicity mechanisms. To investigate whether these mechanisms are involved in the anti-mycobacterial activity of Vγ9Vδ2 T cells, we respectively analyzed the expression of cytotoxicity-related factors, including granzyme A (GrzA), granzyme B (GrzB), perforin (PRF), and granulysin (GNLY), in cell supernatants collected at 48 h and 72 h post-infection using a CBA assay.

**Figure 5:**
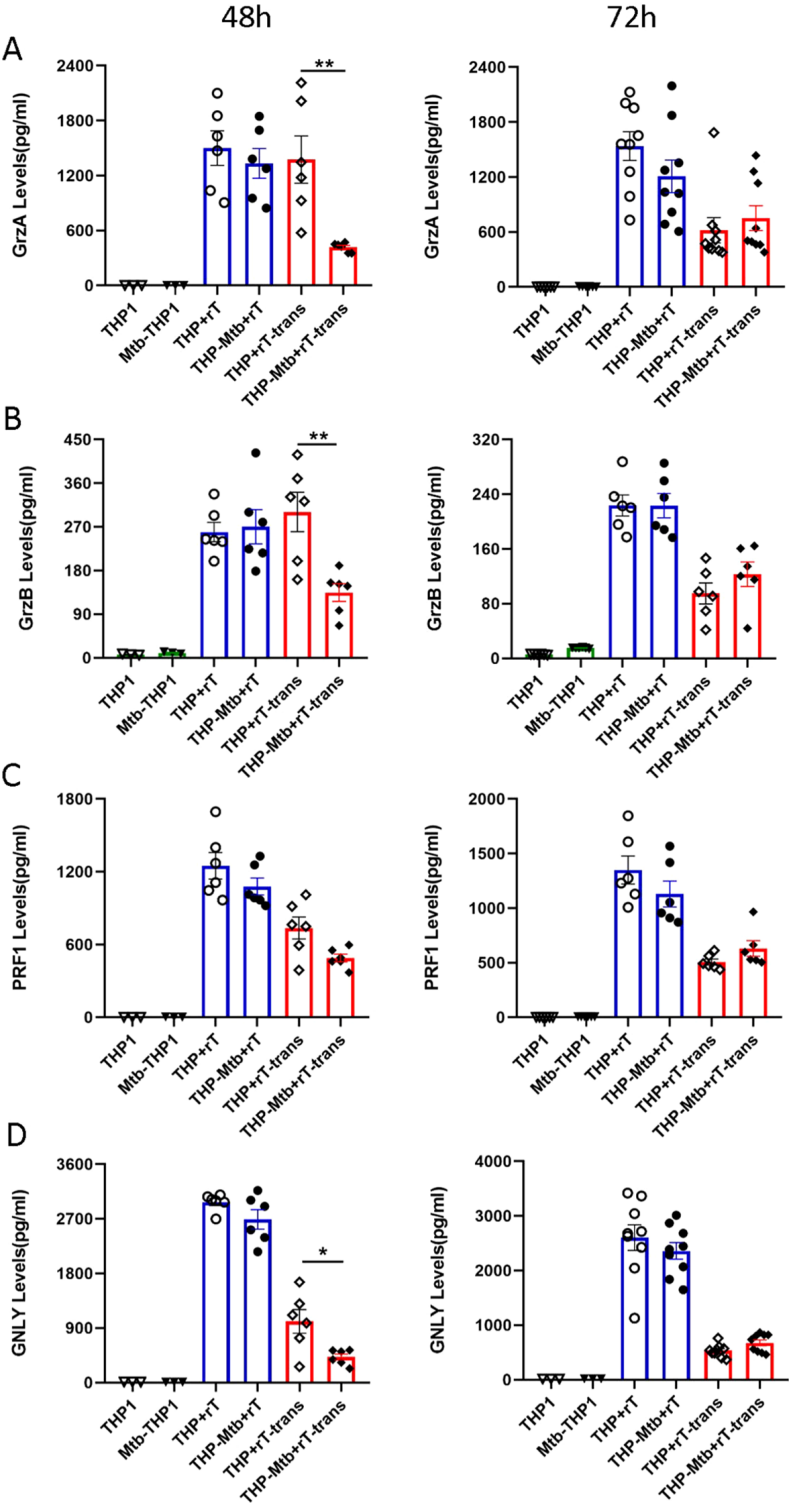
Vγ9Vδ2 T cells expanded with HMBPP, VC, and rIL-2 do not increase the expression of direct cytotoxicity-related factors during inhibition of intracellular mycobacterial infection. Using the CBA, the supernatants from the six cell-infection groups were collected to quantify the levels of cytotoxicity-related factors, including granzyme A (GrzA), granzyme B (GrzB), perforin (PRF), and granulysin (GNLY). The expression levels of **(A)** GrzA, **(B)** GrzB, **(C)** PRF and **(D)** GNLY were assessed in the six groups at 48 and 72h post- infection. The groups included: *THP-1*-derived macrophages (THP1), *Mtb*-infected macrophages (Mtb-THP1), direct contact group of *THP-1*-derived macrophages with Vγ9Vδ2 T cells (THP+rT), direct contact group of *Mtb*-infected *THP-1*-derived macrophages with Vγ9Vδ2 T cells (THP-Mtb+rT), indirect contact group of *THP-1*-derived macrophages with Vγ9Vδ2 T cells using transwell chambers (THP+rT-trans), and indirect contact group of *Mtb*-infected *THP-1*-derived macrophages with Vγ9Vδ2 T cells using transwell chambers (THP-Mtb+rT-trans). Data are presented as mean ± SEM from three independent experiments pooled from 6 healthy donors. Statistical analyses were performed using one-way ANOVA followed by Bonferroni multiple comparison test, with significance defined as *P<0.05, **P<0.01.

No statistically significant upregulation of granzyme A, granzyme B, perforin, or granulysin was observed in the direct co-culture group during *Mtb* infection (**Figures 5A–D**). In the indirect cell contact group, granzyme A, granzyme B, and granulysin levels were significantly downregulated at 48 hours (p < 0.05), with no significant changes observed at 72 hours (**Figures 5A–D**). Although perforin showed a trend toward downregulation (p > 0.05), the difference was not statistically significant during *Mtb* infection (**Figures 5A–D**). Collectively, these findings indicate that the anti-mycobacterial activity of Vγ9Vδ2 T cells expanded with HMBPP, VC, and rIL-2 may be not primarily mediated by direct cytotoxicity mechanisms.

### 3.7 TNF-α and IFN-γ pathways mediate intracellular *Mtb* growth inhibition by Vγ9Vδ2 T cells expanded with HMBPP, VC, and rIL-2

**Figure 6:**
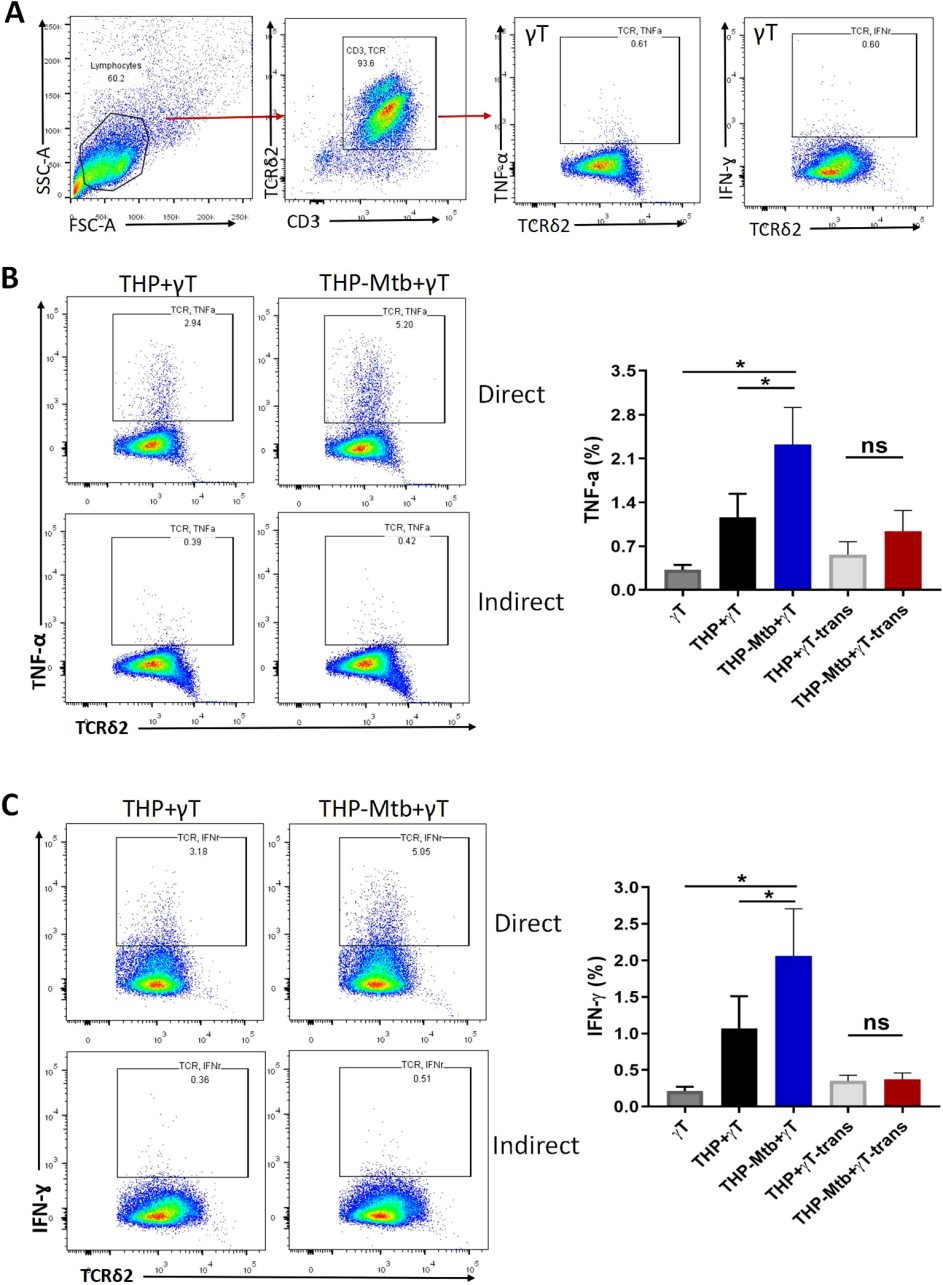
Intracellular expression of TNF- α and IFN-γ is significantly increased inVγ9Vδ2 T cells expanded with HMBPP, VC, and rIL-2 during *Mtb* infection. Purified Vγ9Vδ2 T expanded with HMBPP, VC, and rIL-2 were co-cultured with H37Rv-infeted macrophages for 48h. Six hours before cell collecting, brefeldin A (BFA) and other protein transport inhibitors were added, followed by intracellular staining to assess the expression level of intracellular cytokines. **(A)** Gating strategy of TNF- α and IFN-γ expression in Vγ9Vδ2 T cells. Comparison of the expression levels of **(B)** TNF- α and **(C)** IFN-γ in Vγ9Vδ2 T cells expanded with HMBPP, VC, and rIL-2, either in direct co-culture with H37Rv-infected macrophages or in indirect contact with H37Rv-infected macrophages. The groups included: Vγ9Vδ2 T cells alone cells (γT), direct contact group of *THP-1*-derived macrophages with Vγ9Vδ2 T cells (THP+rT), direct contact group of *Mtb*-infected *THP-1*-derived macrophages with Vγ9Vδ2 T cells (THP-Mtb+rT), indirect contact group of *THP-1*-derived macrophages with Vγ9Vδ2 T cells using transwell chambers (THP+rT-trans), and indirect contact group of *Mtb*-infected *THP-1*-derived macrophages with Vγ9Vδ2 T cells using transwell chambers (THP-Mtb+rT-trans). Data are presented as mean ± SEM from three independent experiments (n=6). Statistical analyses were performed using one-way ANOVA followed by Bonferroni multiple comparison test, with significance defined as *P<0.05. ns, no significant.

Using intracellular cytokine staining and flow cytometry, we analyzed the intracellular expression of TNF-α and IFN-γ in Vγ9Vδ2 T cells expanded with HMBPP, VC, and rIL-2 during *Mtb* infection. The gating strategy for intracellular cytokine analysis is shown in **Figure 6A**. Our results indicated thatVγ9Vδ2 T cells significantly upregulated the expression of TNF-α and IFN-γ during *Mtb* infection, particularly under direct cell-to-cell contact conditions (**Figures 6B, C**, p < 0.001). These findings indicate that the TNF-α and IFN-γ signaling pathways are critical mediators of the anti- mycobacterial activity exerted by Vγ9Vδ2 T cells expanded with HMBPP, VC, and rIL-2.

## 4 Discussion

Vitamin C, a potent antioxidant and a cofactor for enzymes such as histone demethylases and methylcytosine dioxygenases (Xu et al., 2021), exists in two redox states: the reduced active form (ascorbic acid) and the oxidized form (dehydroascorbic acid, DHA). At micromolar concentrations, VC neutralizes reactive oxygen species (ROS), protecting cells from oxidative stress (Corti et al., 2010). At high concentrations, VC induces ROS-mediated cell death in tumor cells, which exhibit high expression of GLUT1, a transporter facilitating DHA uptake. Furthermore, VC modulates multiple pathways involved in immune cell activation and differentiation. For instance, it promotes the development of double-positive T cells from the hematopoietic progenitors and enhances T cell maturation, processes dependent on its antioxidant activity (Manning et al., 2013; Huijskens et al., 2014; Oyarce et al., 2018). VC also stabilizes the regulatory function of induced CD8+ regulatory T cells (Tregs) through FOXP3 demethylation (Iamsawat et al., 2019) and enhances dendritic cell maturation and antigen presentation via the NF-κB and TET-dependent epigenetic regulation (Morante-Palacios et al., 2022). Additionally, VC supports NK cell development from hematopoietic progenitor cells and regulates the methylation status of KIR genes on human NK cells (Wu et al., 2020). Recent studies highlight its role in promoting plasma cell differentiation and enhancing antibody responses throughTET2/3-mediated epigenetic remodeling (Qi et al., 2020; Chen et al., 2022).

VC also enhances the functionality of human γδT cells. It increases Ca^2+^ influx in Zol-expanded γδT cell lines upon anti-CD3 antibody stimulation (Kabelitz et al., 2024) and improves cytokine production, cell cycle progression and metabolic activity (Kouakanou et al., 2020). In our study, VC (70μM) significantly boosted the induction rate of Vγ9Vδ2 T cells to 70% by day 14, with total cell numbers markedly higher than in VC-free cultures (**Figures 1D, E**). These findings suggest that VC enhances the proliferation of Vγ9Vδ2 T cells in healthy PBMCs activated by primary HMBPP and rIL-2 after 12–14 days. Kouakanou et al. demonstrated that VC rescues γδ T cells from activation-induced cell death during restimulation with pAg (Janssen et al., 1991; Kouakanou et al., 2020).

Although Xu et al. reported that VC enhances Vγ9Vδ2 T cell proliferation in Zol-stimulated PBMCs and improves immune effector functions, including proliferation, differentiation and cancer cell killing, they did not observe increased Vγ9Vδ2 T cell expansion in PBMCs primary stimulated by HMBPP (Xu et al., 2021). We speculate that this discrepancy may be attributed to their shorter observation period (8 days). In this study, we extended the induction period to over 14 days and demonstrated that VC significantly enhances the expansion of Vγ9Vδ2 T cells in PBMCs primarily stimulated by HMBPP.

Moreover, Vγ9Vδ2 T cells expanded with HMBPP, VC, and rIL-2 exhibit effector memory phenotypes by day 15 of induction, with 75-90% of these cells expressing the CD45RA^−^CD27^−^ effector memory phenotype. This phenotype is notably decreased in cases of active TB and TB/HIV coinfection (Gioia et al., 2002). Additionally, 10-20% of these cells display the CD45RA^−^CD27^+^ central memory phenotype. In macaques, a robust memory-type response of Vγ9Vδ2 T cells was observed as early as four days after BCG re-infection, with the magnitude of this expansion being 2–9 times greater than that during primary BCG infection (Shen et al., 2002). Furthermore, emerging research suggests that VC enhances the functional efficacy of expanded Vγ9Vδ2 T cells, potentially through mechanisms such as promoting nuclear translocation of NF-κB and NFAT, altering transcriptional profile, enhancing cytotoxic activity, and improving the efficiency of lentiviral transduction (Kabelitz et al., 2024).

On the other hand, Vγ9Vδ2 T cells expanded with HMBPP stimulation are *Mtb*-specifical Vγ9Vδ2 T cells and play a crucial role in inhibiting intracellular mycobacterial growth. To explore this further, we investigated whetherVγ9Vδ2 T cells expanded with HMBPP, VC, and rIL-2 could inhibit the growth of intracellular *Mycobacterium tuberculosis*. Our findings demonstrated that these cells not only inhibit the growth of attenuated strain (H37Ra-mcherry), but also efficiently suppress the growth of high-virulence strain (H37Rv), with the inhibition dependent on cell-to-cell contact. This aligns with previous reports by Daniel F. Hoft, who found that only Vγ9Vδ2 T cells generated through repeated stimulation with BCG-infected DC could directly inhibit intracellular bacilli, achieving an inhibition rate of approximately 50-60% (Abate et al., 2016). Similarly, Chen ZW’s group reported that Vγ9Vδ2 T cells expanded with HMBPP and IL-12 efficiently inhibit intracellular BCG growth in human *THP-1*-induced macrophages and autologous human monocyte-derived macrophages, although their study did not address the inhibitory effects on highly virulent strain (Yang et al., 2019). In our study, we observed that Vγ9Vδ2 T cells expanded with HMBPP, VC and rIL-2 have the ability to inhibit the virulent *Mtb* H37Rv strain, and the inhibition ratio is about 40-60%. We speculate that VC may play a critical role in this process through mechanisms that are yet to be elucidated.

To investigate the mechanism underlying Vγ9Vδ2 T cell-mediated inhibition of intracellular *Mtb* growth, we analyzed the expression of cytokines and cytotoxic factors in co-cultured supernatants using CBA assay. The results showed that both the pro-inflammatory factor IL-6 and the anti-inflammatory factor IL-10 were significantly upregulated during *Mtb* infection. However, the magnitude of IL-6 and IL-10 induction was markedly weaker compared to infections with fast-growing bacteria, such as *Mycobacterium smegmatis* (data not shown). Interestingly, Vγ9Vδ2 T cells expanded with HMBPP, VC, and rIL-2 downregulated IL-10 expression in direct co-culture system while significantly upregulated of IL-6 in the indirect co-culture conditions. This suggests that expanded Vγ9Vδ2 T cells enhance, rather than suppress, the inflammatory response during *Mtb* infection—a finding consistent with Liu et al.’s report that *Mtb* employs a “diplomatic strategy” to establish persistent intracellular infection by balancing bacterial replication and modulating host inflammatory responses (Chai et al., 2019).

**Figure 7:**
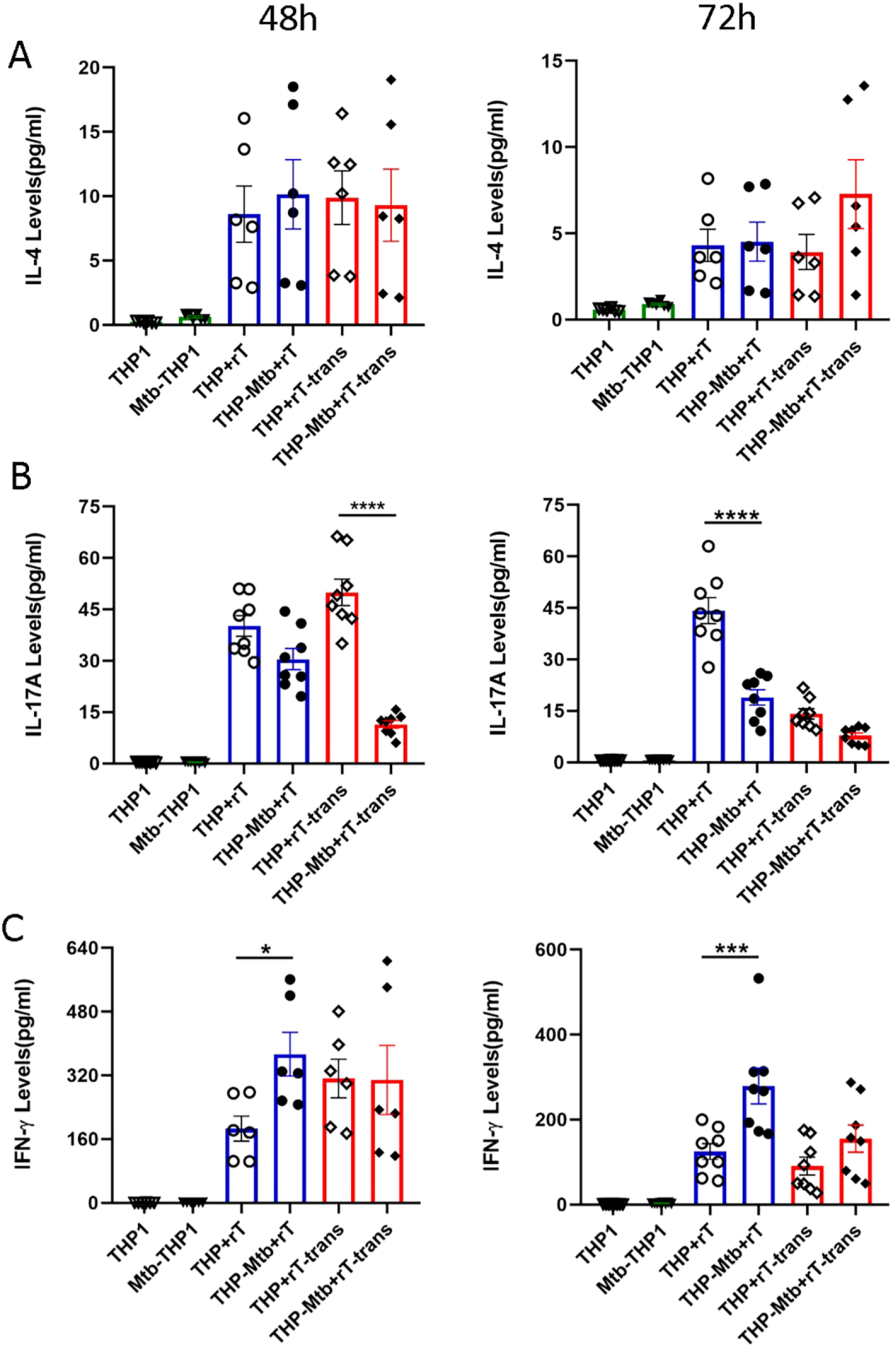
Vγ9Vδ2 T cells expanded with HMBPP, VC, and rIL-2 downregulate IL-17A levels but upregulate IFN-γ expression during mycobacterial infection. Using the CBA, the supernatants from six experimental groups were collected to quantify cytokine levels primarily secreted by Vγ9Vδ2 T cells, including IL-4, IL-17A and IFN-γ. The expression levels of **(A)** IL-4, **(B)** IL-17A, **(C)** IFN -γ were assessed in the six groups at 48h and 72h post-infection. The groups included: *THP-1*-derived macrophages (THP1), *Mtb*-infected macrophages (Mtb-THP1), direct contact group of *THP-1*-derived macrophages with Vγ9Vδ2 T cells (THP+rT), direct contact group of *Mtb*-infected *THP-1*-derived macrophages with Vγ9Vδ2 T cells (THP-Mtb+rT), indirect contact group of *THP-1*-derived macrophages with Vγ9Vδ2 T cells using transwell chambers (THP+rT-trans), and indirect contact group of *Mtb*-infected *THP-1*-derived macrophages with Vγ9Vδ2 T cells using transwell chambers (THP-Mtb+rT-trans). Data are presented as mean ± SEM from three independent experiments pooled from 6 healthy donors. Statistical analyses were conducted using one-way ANOVA followed by Bonferroni multiple comparison test. *P<0.05, ***P<0.001, ****P<0.0001.

The detection results further indicated that TNF-α was significantly up-regulated in the co-culture system, originating from both macrophages and Vγ9Vδ2 T cells regardless of whether it was in the cell-contact group or the non-contact group (**Figure 4C**). However, the significant increase in IFN-γ levels mainly depends on cell-to-cell contact during *Mtb* infection (**Figure 7C**). Using flow cytometry, we measured TNF-α and IFN-γ levels in Vγ9Vδ2 T cells co-cultured with *Mtb*-infected macrophages, and the results revealed a significant upregulation of both cytokines in Vγ9Vδ2 T cells during *Mtb* infection (**Figure 6B**). Vγ9Vδ2 T cells expanded with HMBPP, VC, and rIL-2 significantly downregulated IL-17A expression, modulated IFN-γ levels and had no effect on IL-4 levels during the inhibition of intracellular *Mtb* growth (**Figures 7A–C**).

Previous study has demonstrated that *Mtb* infection leads to a significant increase in TNF-α levels in both alveolar cells and alveolar macrophages (AMs). TNF-α has been suggested to play a pivotal role in regulating AM proliferation and apoptosis during *Mtb* infection (Shi et al., 2016). Furthermore, anti-TNF-α therapy impairs the host defense against infection, particularly by disrupting granulomas formation and maintenance (Athimni et al., 2022). Expanded Vγ2Vδ2 T cells in macaques can differentiate into effector cells, which exhibit the ability to inhibit *L. monocytogenes* infection. This process is associated with elevated levels of cytokines such as IFN-γ, TNF-α, IL-4, IL-17, and effector molecules like perforin (Frencher et al., 2014). Our observation of TNF-α and IFN-γ upregulation under direct co-culture conditions highlights the importance of cell-to-cell contact in potentiating these pro-inflammatory cytokines. This is consistent with the known role of TNF-α in inducing macrophage autophagy and IFN-γ in enhancing phagosome-lysosome fusion, both critical for Mtb clearance (Yang et al., 2019). These findings suggest that *in vitro*-expanded Vγ9Vδ2 T cells may utilize TNF-α and IFN-γ signaling pathways to enhance the phagocytic activity of macrophages and suppress intracellular bacterial growth.

IL-17A is primarily produced by Th17 cells and serves as an early initiator of inflammatory responses. Previous studies have shown that severe TB in juvenile rhesus monkeys downregulates many genes in the blood but upregulates selected genes associated with Th17 and Th1 responses in pulmonary tissues (Qiu et al., 2008). Additionally, Th17-related cytokines contribute to the recall-like expansion and effector function of HMBPP-specific Vγ2Vδ2 T cells following *Mtb* infection or vaccination (Shen et al., 2015). The dual production of IL-17 by multifunctional Th17 cells may correlate with disease severity. Furthermore, circulating levels of IL-17 and IFN-γ are lower in active TB patients than in those with latent TB infection, suggesting that IL-17 deficiency may either predispose individuals to active TB or result from it (Coulter et al., 2017).

Our results demonstrated that Vγ9Vδ2 T cells expanded with HMBPP, VC, and rIL-2 did not significantly alter the expression of granzyme A, granzyme B, perforin and granulysin during the inhibition of intracellular *Mtb* infection. Previous studies have demonstrated that the CD8+ CTL subset can more efficiently eliminate intracellular *Mtb* compared to other subsets through the expression of granzyme B, granulysin, and perforin (Dieli et al., 2001; Balin et al., 2018). Furthermore, we observed that the expression levels of granzyme A, granzyme B, perforin and granulysin in the indirect contact group were downregulated to varying degrees at 48 h post-infection. Daniel F. Hoft et al. noted that serum levels of granzyme A in TB patients were significantly lower than those in individuals with latent TB infection. Purified granzyme A alone has been shown to inhibit intracellular mycobacteria, whereas the knockdown of granzyme A in Vγ9Vδ2 T cells abolished these inhibitory effects (Spencer et al., 2013). These findings collectively indicate that cytotoxic molecules (e.g., granzyme A, granzyme B, perforin and granulysin) may play critical roles in the anti-tuberculosis immune response.

Moreover, granulysin has been demonstrated to eliminate *Mtb* at concentrations higher than those typically found in serum, suggested that additional serum components may also contribute to *Mtb* eradication (Di Liberto et al., 2007). However, no obvious changes in granulysin levels in Vγ9Vδ2 T cells were observed before and after *Mtb* infection. We speculate that Vγ9Vδ2 T cells expanded with HMBPP, VC, and rIL-2 may employ alternative mechanisms to inhibit intracellular *Mtb* growth, such as enhancing the phagocytic and clearance abilities of macrophages or activating bypass signaling pathways. Given that *Mtb* is a slow-growing bacterium, with relatively limited antigen exposure, it elicits a weak immune response in Vγ9Vδ2 T cells and does not significantly activate apoptosis or direct killing pathways. This hypothesis warrants further investigation and discussion.

However, several limitations should be acknowledged. First, the mechanisms underlying VC-mediated enhancement of Vγ9Vδ2 T cell expansion and effector function remain incompletely understood, requiring further mechanistic investigation. Furthermore, the contributions of other cytokines (e.g., IL-22) and immune cell subsets (e.g., NK cells) to this process were not investigated and merited future exploration.

In conclusion, this study demonstrates that VC enhances the *in vitro* expansion of Vγ9Vδ2 T cells primed with HMBPP from healthy donor PBMCs. HMBPP/VC/rIL-2-expanded Vγ9Vδ2 T cells inhibit intracellular *Mtb* growth via TNF-α/IFN-γ signaling pathways. The findings provide a novel immunotherapeutic strategy for tuberculosis. Further optimization of expansion protocols and *in vivo* validation in preclinical models are critical steps toward clinical translation.

## Data Availability

The original contributions presented in the study are included in the article/**Supplementary Material**. Further inquiries can be directed to the corresponding authors.
