# Supplementary material for: Vγ9Vδ2 T cells expanded with vitamin C combined with HMBPP in vitro inhibit intracellular Mycobacterium tuberculosis growth
Source: Front Cell Infect Microbiol. 2025 Apr 30;15:1533277. doi: 10.3389/fcimb.2025.1533277 (PMC12075132; doi:10.3389/fcimb.2025.1533277)
Supplement: Supplementary file 1 [file DataSheet1.pdf]

Vγ9Vδ2 T cells expanded with Vitamin C combined with HMBPP in vitro inhibit intracellular ~~Mycobacterium tuberculosis~~ growth

Supplementary Information

Supplementary Figures: Figure S1-S3

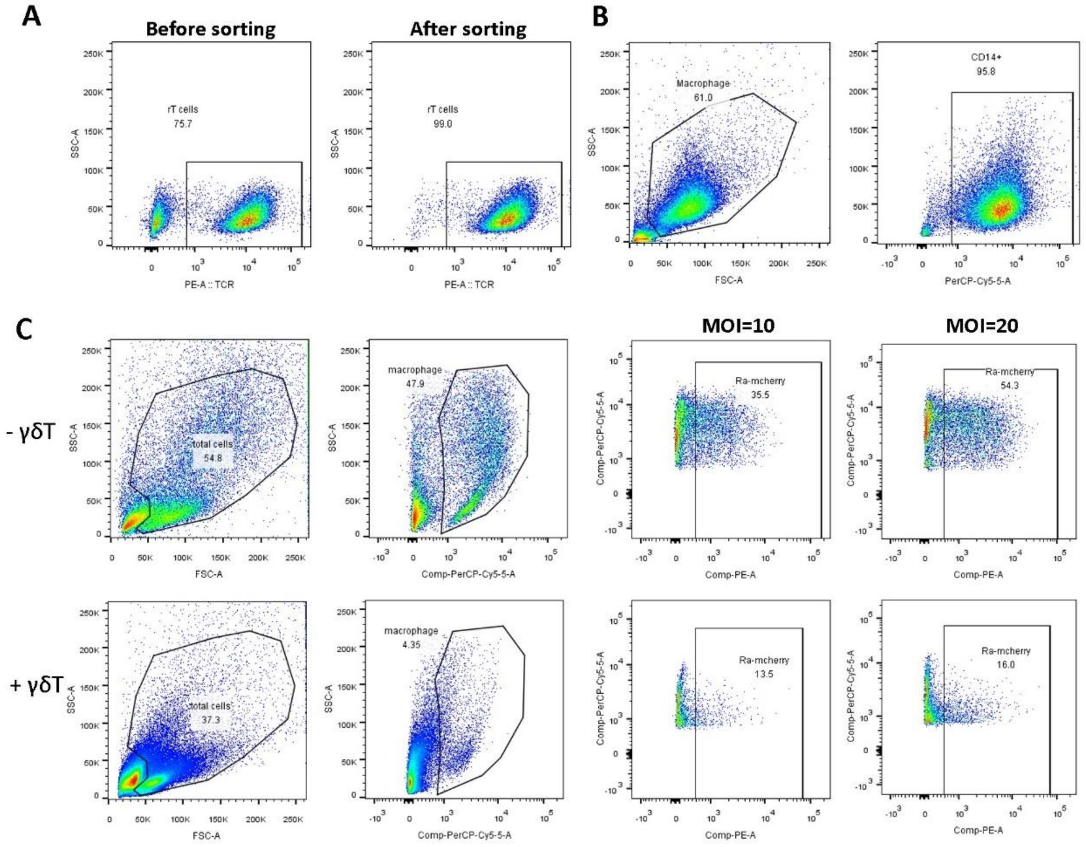

Figure S1. Exploration of the concentration of H37Ra-mcherry infection and the inhibitory effect of Vγ9Vδ2 T cells expanded with HMBPP, VC, and rIL-2 on H37Ra-infected macrophages.

(A) From days 14 to 19, Vγ9Vδ2 T cells expanded with HMBPP, VC, and rIL-2 were sorted using anti-human TCRγ/δ microBeads (Miltenyi Biotec, 130-050-701).

(B) Primary macrophages (CD14+) were derived from healthy PBMCs using a cell adhesion method supplemented with M-CSF.

(C) Primary macrophages were plated in 24-well plates and infected with H37Ra-mCherry strains at MOIs of 10 and 20, respectively. After 4h of adsorption, extracellular *Mtb* was removed. Subsequently, sorted Vγ9Vδ2 T cells were added at an effector-to-target cell ratio of 10:1. Cells were collected at 48h post-infection, and the percentage of macrophages with red fluorescence was quantified by flow cytometry to determine the infection rate. -γδT: H37Ra-mCherry-infected macrophages without Vγ9Vδ2 T cells. +γδT: H37Ra-mCherry-infected macrophages with Vγ9Vδ2

T cells. Data are from three independent experiments involving 3 healthy donors.

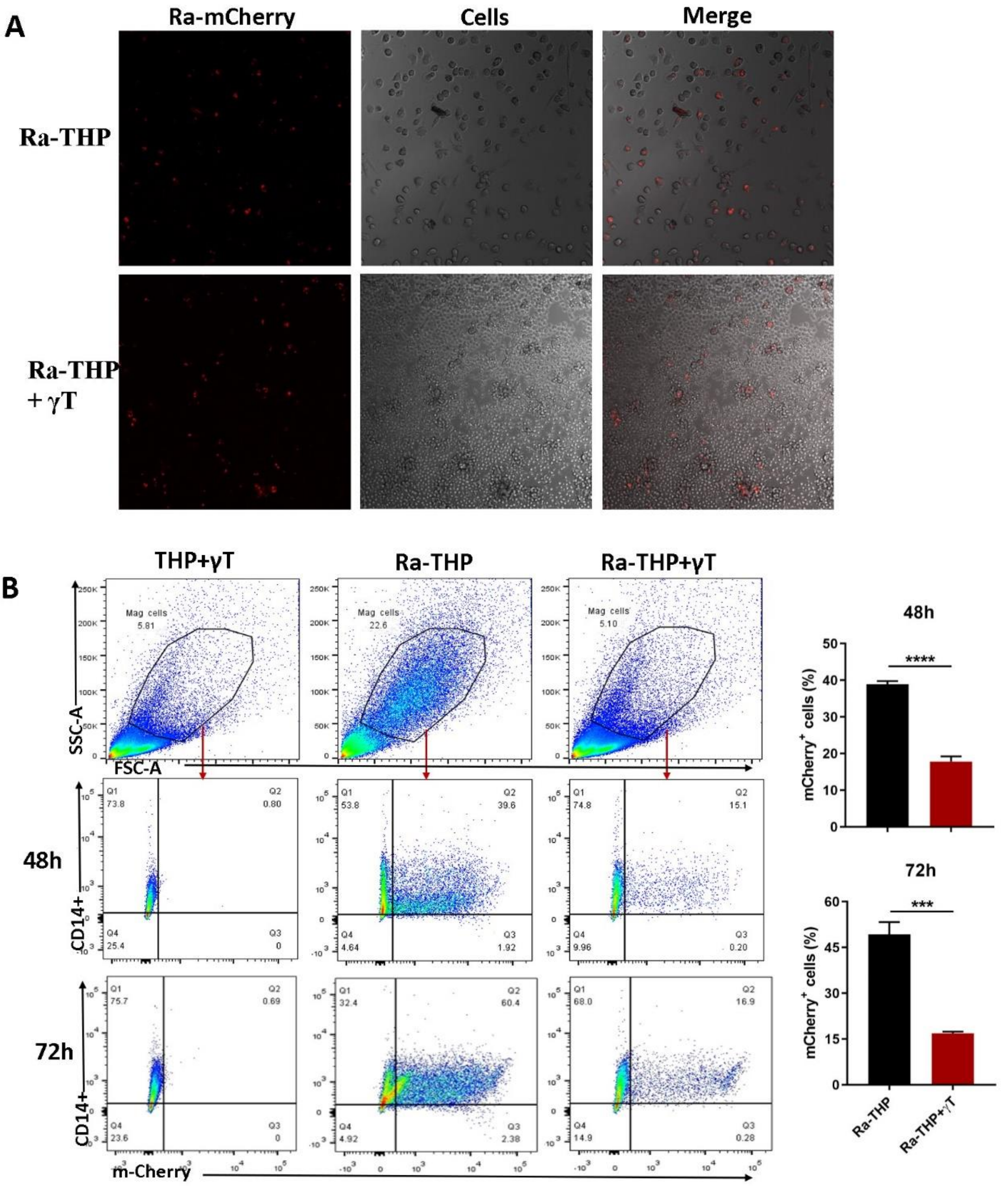

Figure S2. *THP-1*-derived macrophages infected with H37Ra-mCherry were analyzed for *Mtb* infection rates using flow cytometry and laser confocal microscopy

THP1-derived macrophages were infected with the avirulent H37Ra-mCherry strain at MOI of 10. After 4 h of adsorption, purified Vγ9Vδ2 T cells, obtained through magnetic bead sorting, were added to the culture medium of the H37Ra-infected THP1-derived macrophages for co-culture.

(A) Using confocal microscope, the co-localization of macrophages phagocytosing fluorescent bacteria and Vγ9Vδ2 T cells was observed at 24 h post-infection with H37Ra-mCherry strains.

(B) The proportion of PE-positive macrophages among total CD14+cells was analyzed by flow-cytometry, serving as an indicator of the macrophage infection rate with H37Ra at 48 h and 72 h post-infection. Statistical analysis was performed to compare the infection rates of macrophages with and without Vγ9Vδ2 T cells at 48 h and 72 h post-infection. THP+γT: THP-1-derived

macrophages co-culturing with Vγ9Vδ2 T cells. Ra-THP: H37Ra-mCherry-infected THP-1-derived macrophages. Ra-THP+ γT: H37Ra-mCherry-infected THP-1-derived macrophages co-culturing with Vγ9Vδ2 T cells. Data are from three independent experiments (n = 4). Statistical significance was determined using paired t-test analysis, *P<0.05, **P<0.01, ***P<0.001, ****P<0.0001.

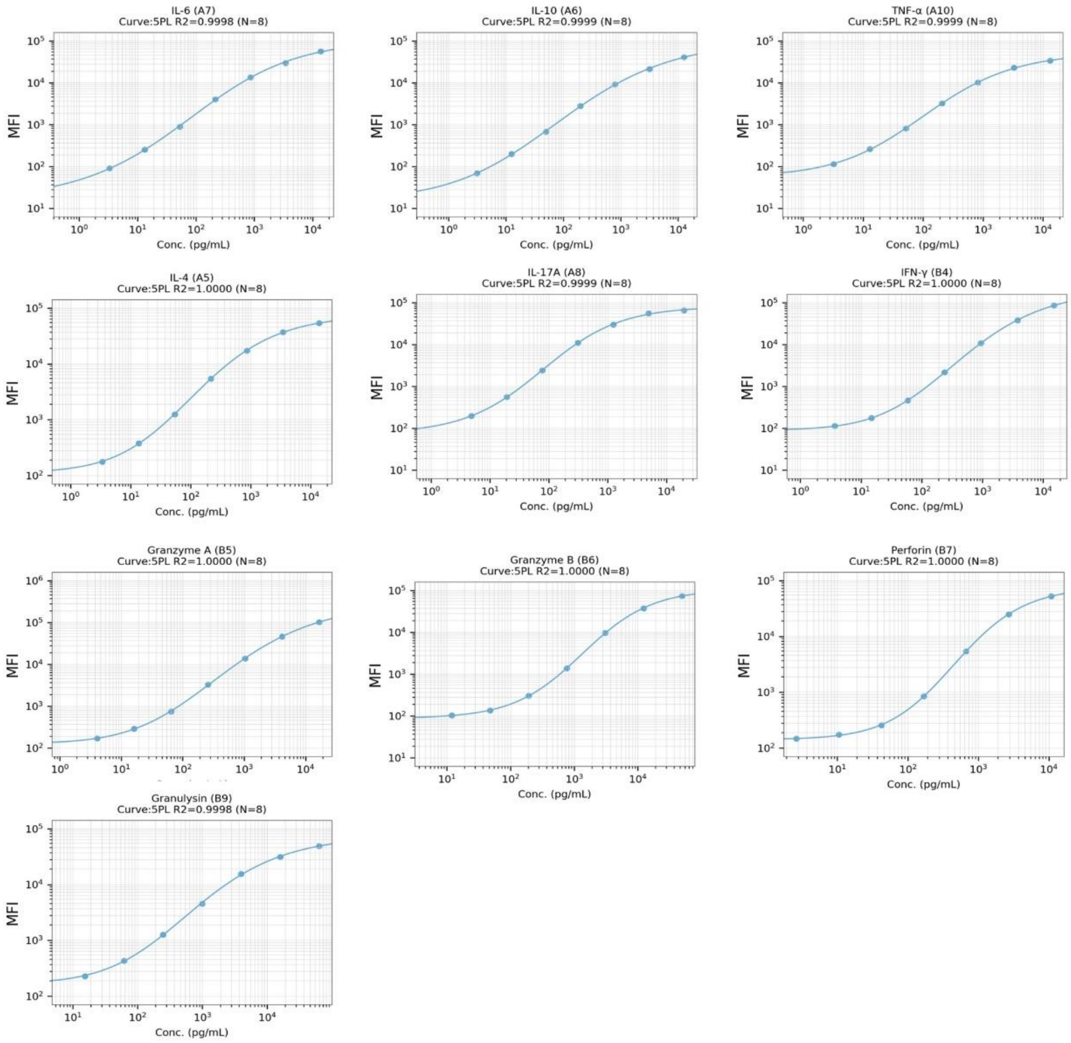

Figure S3. Standard curves for various cytokines.

The standard was dissolved in 250 μL of Assay Buffer and mixed thoroughly by inverting several times. The solution was allowed to stand for 10 minutes and then transferred into a microcentrifuge (EP) tube labeled C7. Seven additional EP tubes were prepared and labeled as C6, C5, C4, C3, C2, C1, and C0, respectively. 75 μL of Assay Buffer was pipetted into to each tube. A 4-fold serial dilution was performed by transferring 25 μL from C7 to C6, and this process was repeated sequentially until reaching C1. Tube C0 contained only Assay Buffer (0 pg/ml). Each concentration gradient was prepared in triplicate, and the average value was calculated. The standard curve was plotted, ensuring a correlation coefficient (R value) of ≥ 0.99 for each cytokine. Detailed procedures were performed in accordance with the reagent manufacturer's instructions.
